# Modulated Electro-Hyperthermia Facilitates NK-Cell Infiltration and Growth Arrest of Human A2058 Melanoma in a Xenograft Model

**DOI:** 10.3389/fonc.2021.590764

**Published:** 2021-02-25

**Authors:** Tamás Vancsik, Domokos Máthé, Ildikó Horváth, Anett Anna Várallyaly, Anett Benedek, Ralf Bergmann, Tibor Krenács, Zoltán Benyó, Andrea Balogh

**Affiliations:** ^1^ 1st Department of Pathology and Experimental Cancer Research, Semmelweis University, Budapest, Hungary; ^2^ Department of Biophysics and Radiation Biology, Semmelweis University, Budapest, Hungary; ^3^ Institute of Translational Medicine, Semmelweis University, Budapest, Hungary

**Keywords:** melanoma, hyperthermia, immunotherapy, NK-cell infiltration, tumor damage

## Abstract

Modulated electro-hyperthermia (mEHT), induced by 13.56 MHz radiofrequency, has been demonstrated both in preclinical and clinical studies to efficiently induce tumor damage and complement other treatment modalities. Here, we used a mouse xenograft model of human melanoma (A2058) to test mEHT (~42°C) both alone and combined with NK-cell immunotherapy. A single 30 min shot of mEHT resulted in significant tumor damage due to induced stress, marked by high hsp70 expression followed by significant upregulation of cleaved/activated caspase-3 and p53. When mEHT was combined with either primary human NK cells or the IL-2 independent NK-92MI cell line injected subcutaneously, the accumulation of NK cells was observed at the mEHT pretreated melanoma nodules but not at the untreated controls. mEHT induced the upregulation of the chemoattractant CXCL11 and increased the expression of the matrix metalloproteinase MMP2 which could account for the NK-cell attraction into the treated melanoma. In conclusion, mEHT monotherapy of melanoma xenograft tumors induced irreversible heat and cell stress leading to caspase dependent apoptosis to be driven by p53. mEHT could support the intratumoral attraction of distantly injected NK-cells, contributed by CXCL11 and MMP2 upregulation, resulting in an additive tumor destruction and growth inhibition. Therefore, mEHT may offer itself as a good partner for immunotherapy.

## Introduction

Modulated electro-hyperthermia (mEHT) is an effective and safe form of hyperthermia, which aims at selective heating of the extracellular matrix and cell membranes in the malignant tissues rather than homogenous heat delivery into tumors (1). The benefits of mEHT as a complementary therapy relies in its property to induce tumor-damage by direct cell killing, mediated by irreversible heat and cell stress and its tolerability by patients as it has almost no side-effects. In the recent years numerous papers have described the growth inhibiting effect of mEHT in various tumor types, mostly in preclinical mouse models (2–6). These results provide evidence that besides the direct cell killing, mEHT has an immunomodulatory effect supporting the anti-tumor immune response (3, 4, 7). In the clinical setting, several case studies have revealed beneficial effects of mEHT (8, 9) including the on-going clinical phase studies which are expected to provide more details on the advantages of combining mEHT with radio- and chemotherapy (10). However, despite evidence supporting the rationale of hyperthermia for augmenting immunotherapy in pre-clinical models, these has not been implemented into the clinical practice (11). Natural killer (NK) cell therapy represents a promising novel treatment modality for both hematological malignancies and solid tumors (12). The cytotoxic activity of the NK cells is independent of tumor antigens, they are tolerant to cells expressing human leukocyte antigen class I (HLA class I) ligands but trigger cytotoxicity against altered cells of reduced HLA-I expression and, due to their short lifetime, the side effects of NK cell immunotherapy are negligible. Clinical trials have been performed with either primary NK cells derived from peripheral blood mononuclear cells (PBMCs) or commercially available clonal NK cell lines, which are easy to expand and maintain *in vitro* (12). The therapeutic outcomes of the adoptive transfer of NK cells was successful mainly in hematological malignancies, while in the case of solid tumors it has been disappointing due to impaired trafficking, infiltration and the immunosuppressive environment of the tumors (13). Several strategies have been proposed to overcome these obstacles and to augment NK cell activity in solid tumors (14). The treatment of NK cells with IL-15 helped to maintain anti-tumor activities in the context of an immunosuppressive microenvironment compared with IL-2 treated NK cells (15). Arming of NK cells with additional CXCR receptors to facilitate their migration toward various cytokine producing tumors (16) or engineering NK-92 cells to express T-cell receptors with tumor antigen specificity have also been proposed as promising strategies in different tumor models (17). In a recent study Yang et al. has reported that the focused ultrasound enhanced the accumulation of NK cells in ovarian cancer xenograft mainly by inducing CX3CL1 expression (18).

The effect of hyperthermia on NK cell mediated anti-tumor response has been extensively studied and reviewed (19). While the *in vitro* hyperthermia diminished the viability and cytotoxic activity of isolated NK cells (19, 20), *in vivo* treatment supported NK cell activity in several tumor models including the very first report about whole body hyperthermia on NK cell cytotoxicity in a patient treated for Ewing’s sarcoma. Hyperthermia was shown to restore and enhance the NK cell activity possibly *via* inducing supportive interferon production (21). Ostberg et al. demonstrated that besides the NK activating pyrogenic cytokines (TNF-α, IFN-γ) secreted during hyperthermia, another possible mechanism behind enhanced NK cell cytotoxicity by fever range thermal stress is associated with plasma membrane NKG2D clustering and increased expression of MICA on target cells (22). Multhoff et al. reported recently that hyperthermia-induced hsp70 promoted NK cell activation, when used in combination with PD-1 inhibition, significantly increased the overall survival in preclinical models of glioblastoma and lung cancer (23, 24). The effectiveness of hyperthermia in melanoma treatment was demonstrated by us and others in several preclinical models (2, 25). Regarding its clinical application Overgard et al. reported that in a phase III clinical trial hyperthermia augmented significantly the fractionated radiotherapy (26).

In the present study we aimed at elucidating the effect of mEHT on A2058 human melanoma xenografts combined with adoptive transfer of primary or immortalized human NK-92MI cells. We demonstrate that mEHT, besides its tumor growth inhibiting effect, augments NK cell infiltration into the treated tumors and thus, it is a promising strategy to enhance the effectiveness of adoptive NK cell transfer.

## Material and Methods 

### Cell Culture

A2058 human melanoma cell line originated from the American Type Culture Collection (ATCC; Rockville, MD, USA), a kind gift of Gabor Tigyi, Department of Physiology, UTHSC, Memphis) was maintained in DMEM with 10% fetal bovine serum in a humidified incubator with 5% CO_2_ at 37°C. Primary human NK cells were isolated from PBMCs of a healthy donor by a density gradient with Ficoll-Paque Plus (Sigma-Aldrich; St. Louis, MO, USA) followed by purification using an NK cell isolation kit (Miltenyi Biotec; Teterow, Germany). Purified NK cells were expanded during 7 days in NK MACS medium (Miltenyi Biotec) supplemented with 2% human serum (Sigma-Aldrich) and 500 U of IL-2 (Miltenyi Biotec) under standard culture conditions in a humidified incubator. NK-92MI cell line purchased from ATCC was grown in NK MACS medium supplemented with 2% human serum and 10 U of IL-2. Cell staining of the NK cells for *in vivo* tracing was done with CellTrace Far Red Cell Proliferation Kit (Thermo Fisher Scientific; Waltham, MA, USA) as described by the manufacturer. For the co-culture experiments A2058 melanoma cells were plated in 12-well plates at 1 x 10^5^/ml in the culture medium described above. Next day graded doses of human primary NK cells were added to A2058 culture to reach the following ratio of 1:0, 1:0.25, 1:0.5, and 1:1 melanoma: NK cell and cultured for two h. Phase contrast microscopy images were taken with the Nikon TI2E microscope using the 20X objective.

### *In Vitro* mEHT Treatment

Sub‐confluent cell-cultures were resuspended in fresh medium at 1 × 10^6^ cells/ml density, then 1.5 ml cell suspension was used for each experimental group. Normothermic controls were treated in water bath for 30 min at 37°C, while 42°C treatment of mEHT was performed between two plan‐parallel electric condenser plates using the Lab‐EHY 100 device (Oncotherm Ltd; Budaörs, Hungary). Right after the treatment cells were cultured for 24 h at 1 x 10^6^ in a 60 mm diameter Petri dish or at 1.25 × 10^5^ cells/well in a 6 well plate for RNA isolation or flow cytometry respectively.

### Quantitative RT‐PCR

Total RNA was extracted 24 h after treatment of tumor cells using the NucleoSpin RNA Plus XS (Macherey-Nagel GmbH & Co. KG; Düren, Germany). Complementary DNA (cDNA) synthesis was done with RevertAid First Standard cDNA Synthesis Kit (Thermo Scientific). The primer sets used were as follows: RPS13 Fwd: CGAAAGCATCTTGAGAGGAACA, Rev: TCGAGCCAAACGGTGAATC, CXCL-9 Fwd: AGTGCAAGGAACCCCAGTAG, Rev: AGGGCTTGGGGCAAATTGTT, CXCL-10 Fwd: AGCAGAGGAACCTCCAGTCT, Rev: AGGTACTCCTTGAATGCCACT, CXCL-11 Fwd: GAGTGTGAAGGGCATGGCTA and Rev: ACATGGGGAAGCCTTGAACA (Merck KGaA; Darmstadt, Germany). Quantitative real-time PCR was performed with CFX Connect Real‐Time PCR Detection System (Bio-Rad; California, USA) using the SsoAdvanced Universal SYBR Green Supermix (Bio-Rad). RPS13 was used as internal control, and the fold change in expression was calculated as 2^−ΔΔCT^.

### Flow Cytometry

Dying cell fractions were identified and measured by flow cytometry. 24 h post-treatment the supernatants were collected and cells were trypsinized, then placed on ice for all further steps. The suspensions were centrifuged for 5 min at 300 × g, washed twice in PBS and resuspended in Annexin V Binding Buffer at a concentration of 1 × 10^6^ cells/ml. To 100 µl of cell suspension 2.5 µl Alexa Fluor^®^ 647 Annexin V stock solution (Biolegend; San Diego, CA, USA) and 1 µl of 1 mg/ml propidium‐iodide (Sigma‐Aldrich) was added. Samples were incubated in dark on ice for 20 min, then further diluted with 200 µl Annexin V Binding Buffer (Biolegend) and measured by flow cytometry. 638 nm wavelength excitation laser was used for Alexa Fluor^®^ 647 Annexin V and 488 nm for PI fluorochrome. Based on its low forward and side scatter (FSC/SSC) properties, cell debris was excluded from the measurement, where 2 × 10^4^ events per sample were counted by using flow cytometry (CytoFLEX Flow cytometer, Beckman Coulter Life Sciences; Indianapolis, USA). Obtained data were analyzed with CytExpertCell software (Beckman Coulter Life Science). For granzyme B detection primary NK cells were stained with FITC-labeled anti-human/mouse granzyme B antibody (#505403, Biolegend) according to the manufacturer’s protocol. Cells were measured using 488 nm blue laser excitation wavelength and 525/40 nm bandpass filter for detection.

### *In Vivo* mEHT Treatment Model and *In Vivo* Imaging

The A2058 tumor cell suspension (1 million cells/100 µl) was inoculated subcutaneously into both flanks of NOD.CB-17-Prkdc scid/Rj mice (Janvier Labs; Le Genest-Saint-Isle, France). Depending on the follow-up timepoint different tumor sizes were used. When mice were sacrificed 24 post-treatment in order to characterize the early stress response, the tumors were treated when having 10 mm diameter while when the animals were used for 4-day follow-up the xenografts had 5-6 mm diameter (0.1–0.15 cm^3^ volume).

Once the tumor had reached the desired volume, the right-side tumors were subjected to mEHT or sham treatment and the left-side ones were left untreated and used as controls. The mEHT treatment was performed as described before (2) by using the Lab‐EHY 100 device (Oncotherm Ltd, Budaörs, Hungary). In the sham-treated mice the right-side tumors were placed between the electrodes and exposed to the same physical force like the treated ones. Tumor growth was monitored with ultrasound measurement (Philips Sonos 5500 Ultrasound; Amsterdam, Netherlands). Whole body imaging was performed to detect far red fluorescence with the fluorescent optical imaging system FOBI (CELLGENTEK Co., Ltd., Suwon, South Korea). Images were analyzed using VivoQuant software (inviCRO, LLC; Boston, USA). All animal work conducted during this study was approved by the Hungarian National Scientific Ethical Committee on Animal Experimentation under No.PE/EA/51-2/2019.

### Immunohistochemistry

Tumors were fixed in 4°C buffered 8% formalin for 48 h, dehydrated and embedded into paraffin wax. Then, 3 µm thick sections were cut, dewaxed and rehydrated prior to hematoxylin-eosin (H & E) staining or immunohistochemistry (IHC). Endogenous peroxidase activity was blocked for 15 min in methanol containing 3% H_2_O_2_. For antigen retrieval, rehydrated sections were heated at ~105°C in Tris-EDTA (TE) buffer pH 9.0 (0.1 M Trisbase and 0.01 M EDTA) for 20 min in a pressure cooker (2100 Antigen Retrieval, Aptum Biologics Ltd.; Southampton, United Kingdom) followed by 20 min cooling. After blocking non-specific protein-binding sites in 0.1 M Tris-buffered saline (TBS, pH7.4) containing 3% bovine serum albumin (BSA, Millipore; Kankakee, Illinois, USA), 0.1% TWEEN^®^ 20 (Sigma-Aldrich) and 0,01% sodium-azide (BSA/TBST) for 20 min, slides were incubated in a humidity chamber at room temperature using antibodies (listed in **Table 1**) diluted in 1% BSA/TBST. HISTOLS^®^ Labeled polymer-peroxidase (anti-rabbit Ig) detection system (Histopathology Ltd.; Hungary) was applied for rabbit monoclonal antibodies (mAbs) with an additional preliminary incubation step using rabbit anti-mouse Ig for mouse mAbs, for 40 min each. For the immunoperoxidase development a DAB chromogen/hydrogen peroxide kit (Leica-NovoCastra; Newcastle Up-on-Tyne, UK) was used. Samples were washed for 3 × 3 min in TBST (pH 7.4) buffer between the incubations. Stained slides were digitalized and the immunoreactions were evaluated using modules of QuantCenter image-analysis software package (3DHISTECH; Budapest, Hungary). Positive immunoreactions for cleaved caspase-3, hsp70, p53, MMP-2, was determined as percentages within annotated tumor areas (HistoQuant module). In case of cleaved caspase-3 (cC-3), hsp70, p53, p21, and AIF the late apoptotic areas which might be lysed by metalloproteases, were excluded from the evaluation. For p53 and p21 evaluation nuclei with strong positive reaction were masked and calculated its area compared to the whole tumor area.

**Table 1:** Antibodies and conditions used for immunohistochemistry and immunofluorescence.

Antigen	Type	Reference no.	Dilution	Vendor
cC-3	Rabbit, pAb	#9664	1:100	Cell Signaling
Hsp70	Rabbit, pAb	#4872	1:50	Cell Signaling
CD57	Mouse, mAb	#MAD-002084QD–7	RTU	Master Diagnóstica
MMP-2	Rabbit, pAb	# RB-9233-PO	1:50	Neomarkers
p53	Mouse, mAb	#BP-53-12	RTU	Genemed
p21	Rabbit, mAB	#188224	1:500	Abcam
AIF	Rabbit, pAb	#4642	1:25	Cell Signaling

Vendor specification: Cell Signaling (Danvers, MA, USA), Neomarkers (Portsmouth, NH, USA), Genemed Biotechnologies, Inc. (San Francisco, CA, USA), Master Diagnóstica (Granada, Spain), Novus Biologicals (Centennial, CO, USA).

### Statistical Analysis

Statistical analysis was performed using GraphPad Prism software (v.6.07; GraphPad Software Inc.; La Jolla, CA, USA). Statistical significance between groups during the tumor growth follow-up was calculated using two-way ANOVA followed by Tukey’s multiple comparisons. Mann–Whitney nonparametric test was performed for statistical comparison between treated and control groups. In experiments with more than two groups, statistical differences were assessed with ANOVA accompanied by Tukey’s or Sidak’s multiple comparisons test. p-values < 0.05 were considered significant.

## Results

### mEHT Inhibits A2058 Melanoma Tumor Growth

**Figure 1:**
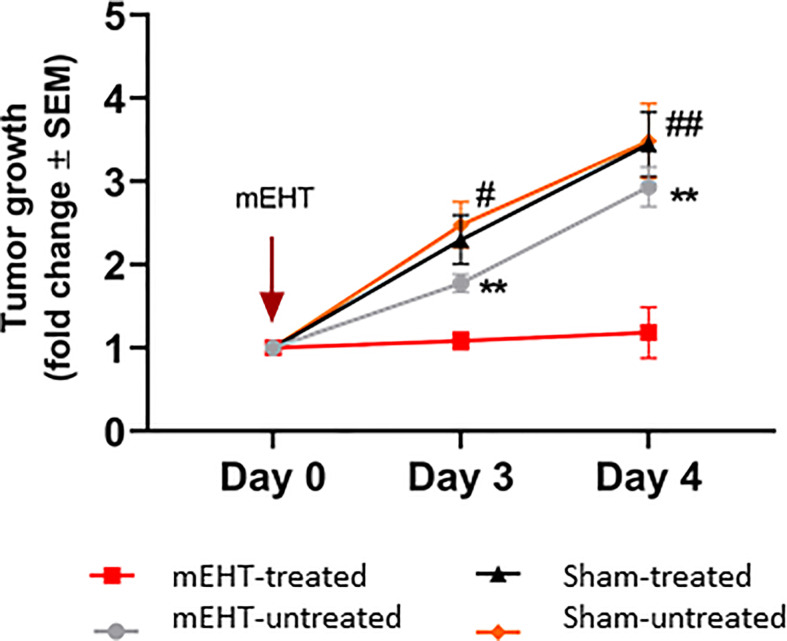
Tumor growth is arrested by modulated electro-hyperthermia (mEHT) treatment. N=6 for mEHT-treated and mEHT-untreated, n=5 for sham-treated and sham-untreated, **p=0.006 between mEHT-treated and untreated, ^#^p=0.01 on day three and ^##^p=0.007 on day four between mEHT-treated and sham-untreated, *p=0.01 for mEHT and sham-treated on day three and four using two-way ANOVA followed by Tukey’s multiple comparisons test.

To examine the effect of mEHT on A2058 melanoma, we have exposed established tumors to a single mEHT or sham treatment lasting for 30 min and monitored the tumor growth up to day four by using ultrasound. Mice were divided in two groups. In one group symmetrically grown tumors were either treated with mEHT or left untreated. In the other group, tumors were sham treated or left untreated. We found that already a single mEHT treatment had a marked effect on tumor growth. There was a significant difference manifested on the third- and fourth-day post treatment both between the mEHT-treated and its untreated counterpart (p=0.006) and also between mEHT-treated and the sham-treated tumors (p=0.01). There was no significant difference between the sham-treated and sham-untreated tumors at any timepoint (**Figure 1**).

### mEHT Activates p53 and Induces Caspase-3 Dependent Cell Death

**Figure 2:**
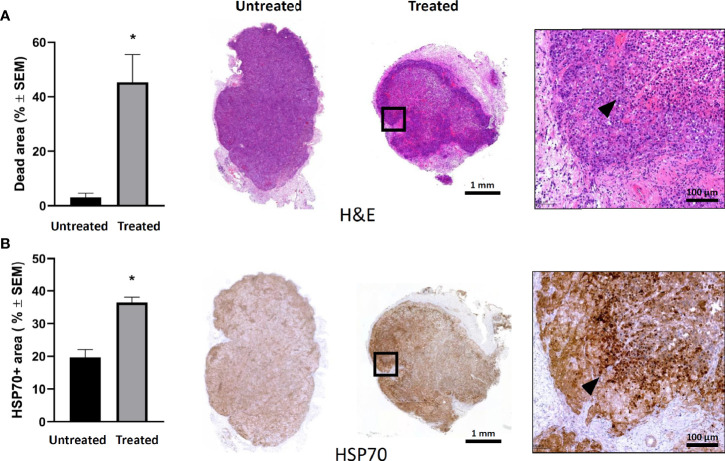
Modulated electro-hyperthermia (mEHT) monotherapy-induced cellular stress results in massive tumor destruction of established human A2058 xenografts**. (A)** Quantitative analysis and representative images of hematoxylin-eosin (H & E) staining reflecting live/death tumor areas of untreated and mEHT-treated tumors. **(B)** Heat stress response demonstrated by hsp70 expression. Arrowheads indicate characteristic staining of damaged tumor tissue and hsp70 expression in the mEHT-treated tumor sections. Samples were analyzed 24 h post-treatment, *p=0.02, n=4. Statistical significance was calculated using Mann-Whitney test.

Histological analysis of the tumors 24 h after a single mEHT treatment showed massive cell death in the treated side (**Figure 2A**). While in the non-treated tumors the percentage of the dead area is negligible, in the treated ones it reached 40% (*p=0.02). The cellular stress induced by mEHT was accompanied by abundant hsp70 expression which doubled one day post-treatment (*p=0.02) (**Figure 2B**).

**Figure 3:**
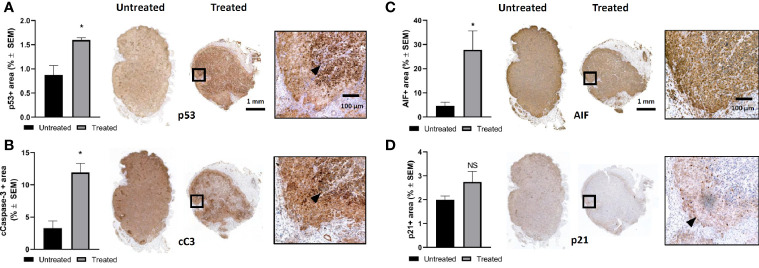
Modulated electro-hyperthermia (mEHT) treatment induces caspase dependent apoptosis *via* p53 activation in established human A2058 xenografts. **(A)** Quantitative analysis and representative images of immunostained tumor sections for p53, *p=0.02, n=4. **(B)** Cleaved caspase-3 (cC-3) *p=0.02, n=4. **(C)** AIF (apoptosis inducing factor) *p=0.04, n=6 and **(D)** D. p21 which is not significantly (NS) altered demonstrating that cellular senescence is not induced by mEHT. Arrowheads point to characteristic expression of the given protein on the mEHT treated tumor sections. Samples were analyzed 24 h post-treatment. Statistical significance was calculated using Mann-Whitney test.

In order to elucidate the cell killing mechanism of mEHT in A2058 melanoma xenografts, we used immunohistochemistry (IHC) to detect the expression of several proteins involved in apoptosis. We reported before that mEHT stabilized and induced the nuclear translocation of p53 in B16F10 mouse melanoma cell line *in vitro* and *in vivo* (2). Regarding the p53 status, A2058 contains *TP53^MUT^* which is still functional as upon exposure to nutlin-3, the prototypic MDM2 antagonist, p53 increased and led to apoptosis (27). Using IHC we detected the expression level of p53 one day post-treatment. Our results demonstrate that in response to mEHT-induced stress the p53 protein level elevated and it was two-fold higher as compared to the non-treated, control side tumors (*p=0.02) (**Figure 3A**). Next, we investigated the expression of p21, the canonical p53 target gene, but it did not show any significant change (**Figure 3D**), which points to the fact that tumor cells at this timepoint (24 h post-treatment) are not in the cell cycle arrest phase but rather underwent apoptosis. To demonstrate this, we measured the level of cleaved caspase-3 and found that it was three times higher in the treated tumors compared to the non-treated counterparts (*p=0.02) (**Figure 3B**). It was shown before that mEHT could activate not only the caspase dependent but also the caspase independent apoptotic pathway characterized by high apoptosis inducing factor (AIF) expression (28). **Figure 3C** illustrates that in A2058, despite the fact that mEHT elicits significant increase of this protein, as its localization remains cytoplasmatic and not nuclear AIF is not contributing to mEHT-induced cellular death.

### mEHT Treatment Facilitates NK Cell Infiltration of Melanoma

**Figure 4:**
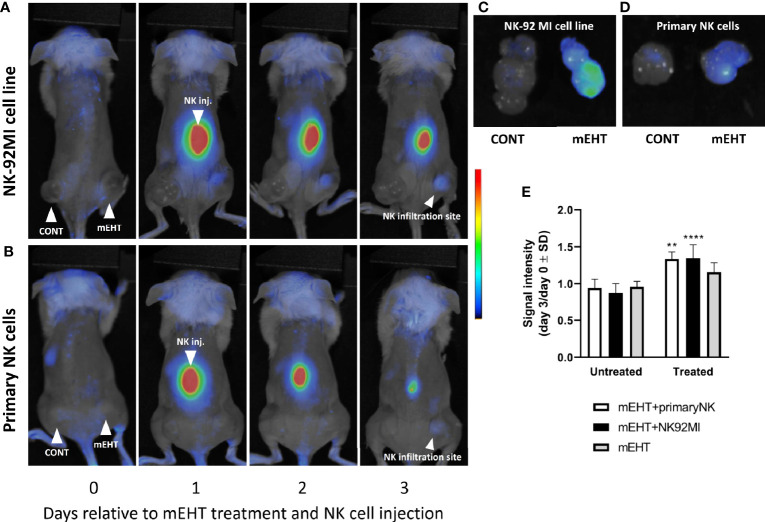
The modulated electro-hyperthermia (mEHT) treatment generated tumor microenvironment favors and permits NK infiltration of melanoma tumors regardless of natural killer (NK) cell origin. **(A, B)** *In vivo* imaging of tumor bearing mice with right side treated and left side control. Fluorescently labeled primary NK cells or NK92MI cells were injected subcutaneously one day post mEHT. *In vivo* imaging was done during 3 days to follow the NK cell trafficking. **(C, D)** Explanted control and mEHT-treated tumors infused by NK-92 MI or primary human NK cells. **(E)** Change of the signal intensity represented as ratio of the fluorescence detected on day 0 and day 3 in control and treated tumors infiltrated with fluorescent NK cells. Significance was calculated using two-way ANOVA followed by Sidak’s multiple comparisons test. n=3 for untreated/treated + primary NK cells, n=6 for untreated/treated +NK92MI, n=5 for untreated/treated mEHT. *p between mEHT-untreated and treated counterparts; **p=0.001 for primary NK cell infused tumors, ****p<0.0001 for NK92MI cell line infiltrated tumors.

We had reported previously that *in vivo* mEHT treatment causes downregulation of MHC-I expression in B16F10 mouse melanoma tumors (2). Our hypothesis was that this tumor phenotype was favorable to the cytotoxic activity of NK cells. Furthermore, the mEHT generated tumor microenvironment is rich in damage associated molecular pattern (DAMP) proteins (2–4), which are known to serve as chemoattractant to facilitate the recruitment of NK cells to the tumor site (4, 25). In the B16F10 mouse melanoma model we could not prove this unequivocally, at least not based on the frequency of the tumor infiltrating NK cells as there was no significant difference between the treated and control tumors. In order to address this question, we designed a different animal model, where we traced the distribution of *in vitro* expanded and fluorescently labeled primary NK cells or the immortalized NK-92MI cell line injected subcutaneously. Similar to the tumor growth model discussed above, we used mice implanted bilaterally and treated the right-side tumor only. One day after the mEHT treatment we injected the labeled NK cells at equal distance from both tumors subcutaneously above the lumbar region of the spine and traced their distribution daily using *in vivo* imaging system. The fluorescence signal was detectable already on day one after NK cell injection and further intensified during the following days within the treated tumor (**Figures 4A, B**). The accumulation of NK cells was assessed based on the ratio of the fluorescence detected on day 0 and day 3 in untreated control and treated tumors. Measured signal on day three was significantly higher when mEHT was followed by adoptive NK cell transfer regardless of NK cell origin (**Figure 4E**), whereas in the untreated tumors low intensity was detected in the explanted tumors only (**Figures 4C, D**).

**Figure 5:**
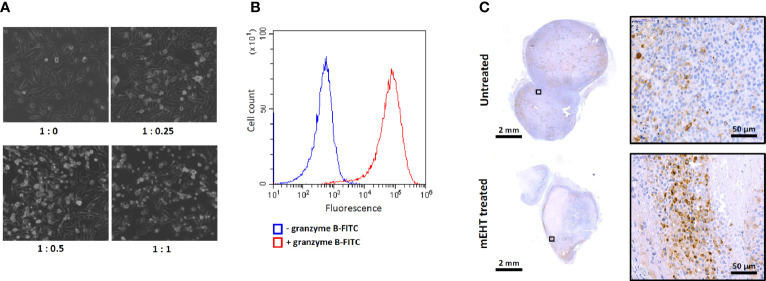
Characterization of ex vivo expanded primary NK cells. **(A)** Cytotoxic effect of primary NK cells and susceptibility of A2058 melanoma to natural killer (NK) cell killing demonstrated in co-culture. Graded doses of NK cells were added to A2058 culture in different ratios, phase contrast microscopy images were taken after 2 h of co-culture. Note the change in morphology of the melanoma culture from adherent to lysed, floating rounded dead cells in the presence of NK cells which indicates that tumor cells might have been eradicated by NK cells. **(B)** Flow cytometry analysis of granzyme B expression in primary NK cells. **(C)** Detection of CD57 positive primary NK-cells in modulated electro-hyperthermia (mEHT) treated and control tumors. Arrowheads emphasize the area where the average abundance of CD57+ marker can be seen on the control and an NK-infiltrated site of the mEHT samples.

To characterize the cytotoxicity of the *ex vivo* expanded primary NK cells and the susceptibility of the A2058 melanoma cell line to NK cell killing we set up an *in vitro* co-culture of the two cell types by adding graded doses of NK cells to A2058 melanoma cells. The phase contrast microscopy images shown on **Figure 5A** demonstrate that our primary human NK cells are potent and strongly cytotoxic and as such they are capable of lysing the A2058 cells. Additionally, the granzyme B expression, what plays crucial role in the cytotoxic effect of the primary NK cells was also measured by flow cytometry and we found that 100% of the population stains positive for this specific marker (**Figure 5B**).

As we demonstrated above, based on the fluorescence signal intensity within *in vivo* conditions, the primary NK cells were able to sense and migrate toward the mEHT treated tumors. The intratumoral presence of the primary NK cells was further confirmed by immunostaining, using the NK specific CD57 marker (**Figure 5C**).

**Figure 6:**
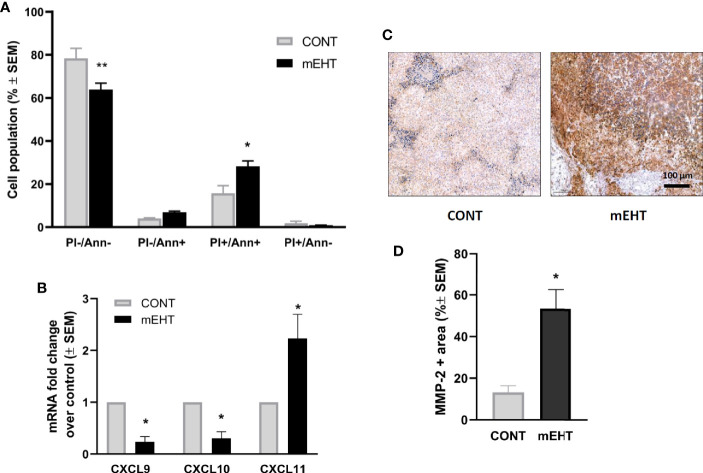
Tumor damage and the potential mechanism of natural killer (NK)-cell trafficking to modulated electro-hyperthermia (mEHT) treated melanoma. **(A)** *In vitro* mEHT treatment induces apoptosis of the A2058 cells marked by the elevated PI+/Ann+ population detected by flow-cytometry 24 h post-treatment, data shows the average of six experiments, *p=0.01, **p=0.003. **(B)** *In vitro* mEHT treatment regulates the expression of chemoattractants in A2058 cells detected by qPCR 24 h post-treatment, data shows the average of three experiments, *p=0.02 calculated with Mann-Whitney test. **(C, D)** mEHT upregulates MMP-2 expression detected by IHC 24 h post-treatment, n=4, *p=0.02 calculated with Mann-Whitney test.

Next, we investigated the potential mechanism by which mEHT could enhance the migration of NK cells with respect to chemokines and MMP-2. The recruitment of NK cells in the inflamed tissues is regulated by the expression of several chemokine receptors, including CXCR3 that binds to the tumor-derived chemokine ligands (29). It has been shown that some of the inflammatory chemokines that recruit NK cells (CXCL9, CXCL10, CXCL11, and CXCL12) contain putative HSF1-binding sites and thus could be upregulated by thermal stress (30). To test whether mEHT can induce the expression of chemokines, we exposed A2058 cells to mEHT *in vitro* and measured the gene expression one day after treatment using qPCR. In order to confirm the effect of mEHT treatment first, we measured the extent of apoptosis using flow cytometry. As expected, mEHT was cytotoxic and resulted in a two-fold increase of the annexin V+ PI+ apoptotic population (*p=0.01) (**Figure 6A**). The qPCR result shows that mEHT had disparate effect on the tested chemokines as out of the three tested chemokines CXCL9 and CXCL10 were downregulated and CXCL11 was upregulated by thermal stress (*p=0.02) 24 h post-treatment (**Figure 6B**). Besides the chemoattractants, the migration of NK cells is determined by its ability to cross the ECM and basement membrane. These structures are degraded by MMPs produced by both the NK cells and the tumor cells. Using IHC we detected the MMP-2 protein expression and we show that one day after treatment the MMP-2 positive area was five times higher in the mEHT treated tumors as compared to the control group (*p=0.02) (**Figures 6C, D**).

### mEHT Induced Tumor Microenvironment Augments NK Cell Cytotoxic Activity

**Figure 7:**
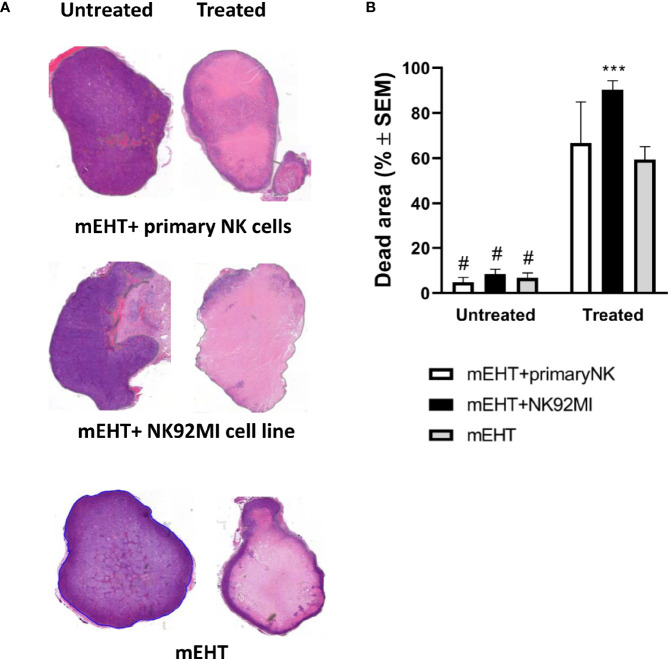
NK cell therapy effectively complements the modulated electro-hyperthermia (mEHT) treatment. **(A, B)** Additive effect of natural killer (NK) cell therapy is demonstrated by hematoxylin-eosin (H & E) staining and evaluation of the damaged tumor area. n=3 for untreated/treated + primary NK cells, n=6 for untreated/treated +NK92MI, n=5 for untreated/treated sham, n=8 for untreated/treated mEHT. Statistical significance was calculated using two-way ANOVA followed by Sidak’s multiple comparisons test. ***p=0.0007 between mEHT +NK92MI cell line infiltrated and mEHT treated tumors and ^#^p <0.0001 between untreated tumors and treated counterparts.

To assess the effect of combined mEHT and NK cell immunotherapy on tumor destruction, the explanted tumors were subjected to histology and immunostaining for cleaved caspase-3. Based on the H & E staining, both in the mEHT and the mEHT combined with adoptive NK cell transfer cases, the treated tumors contain extensive damaged areas (**Figure 7A**). When the comparison was done between treated and untreated counterpart statistical significance was reached in each treatment group. The mEHT monotherapy resulted in massive cell death represented by 60% of the tumor area accounting for the strong statistical significance versus mEHT untreated (^#^p <0.0001). The percentage of damaged tumor area escalated to almost 100% when the treatment was combined with the NK92MI cells and it was statistically significant not only when compared to its untreated counterpart (^#^p <0.0001) but also versus monotherapy (***p=0.0007). When the primary NK cells were administered after the mEHT, we could not detect additive effect of the cell treatment versus the mEHT monotherapy (**Figure 7B**).

**Figure 8:**
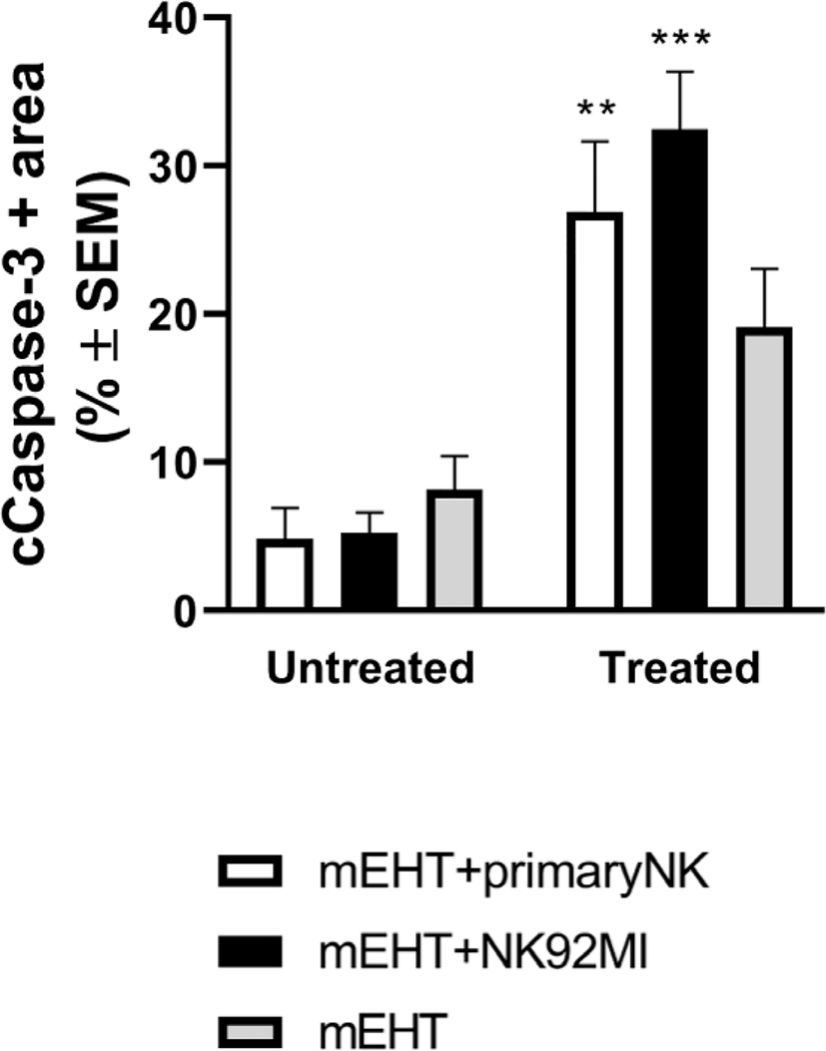
Caspase-3 expression is more increased in tumors treated with modulated electro-hyperthermia (mEHT) combined with primary or NK92MI cell line. Statistical significance was calculated using two-way ANOVA followed by Sidak’s multiple comparisons test. **p=0.003 between mEHT-treated + primary natural killer (NK) cells and untreated + primary NK cells, ***p=0.0005 between untreated mEHT+NK92MI vs treated mEHT+NK92MI cell line.

Next, we characterized more specifically the apoptotic response using cleaved caspase-3 staining (**Figure 8**). As expected, our findings correlate with the previous data: the caspase positive area is the highest in the mEHT+NK92MI treated group with statistical value of ***p=0.0005 between untreated and treated mEHT+NK92MI tumors. Strong cC-3 expression was detected also in the mEHT+primary NK cell-treated group, **p=0.003 between untreated mEHT+primary NK vs treated mEHT + primary NK cells, while no significant difference was noted in the mEHT treated/untreated groups.

## Discussion

In a recently published paper, we described the effect of mEHT on B16F10, a mouse melanoma cell line, *in vitro* and *in vivo* (2, 31). The repeated mEHT treatment resulted in a delayed tumor growth marked by cell cycle arrest and senescence but without considerable immune activation of the host. In the present research, we make use of immunodeficient NOD/SCID mice to extend our studies on the effectiveness of mEHT on human melanoma using the A2058 cell line and, furthermore, to investigate the role of mEHT in combination with NK cell immunotherapy. First, we provided evidence of the cytotoxicity of mEHT on A2058 cells, showing that a single mEHT treatment was sufficient to elicit a significantly delayed growth of established tumors which were more susceptible to the deleterious effect of the treatment than the B16F10 mouse melanoma cell line. Dissecting the mechanism behind this effect we identified several key proteins. Similarly, to B16F10 mouse melanoma tumors, mEHT induced DNA damage and p53 activation but in contrary to B16F10, where this led to cell cycle arrest and senescence marked by high p21^waf1^ expression without pronounced cell death (2) in the A2058 human melanoma xenografts the mEHT-induced p53 expression resulted in strong caspase-3 activation and subsequent apoptosis without significant p21 induction. This observation is in line with previous findings reported by Vancsik et al. (3, 32) who demonstrated that in C26 colorectal carcinoma the *in vitro* mEHT treatment induced p53 activation and caspase dependent cell death similarly to the *in vivo* experiments, where besides the caspase dependent cell death, they also detected the caspase independent pathway, marked by the elevated expression of apoptosis inducing factor (AIF) (28). In our present model the AIF protein was also upregulated by mEHT, but its nuclear translocation was inhibited, probably complexed by hsp70, recapitulating the findings we had described in the case of B16F10 mouse melanoma (2).

In the second part of our study, we investigated the effect of mEHT on the tumor microenvironment with respect to NK trafficking, homing and tumorigenic activity. While several lines of evidence indicate the effect of hyperthermia through hsp70 on the cytolytic activity of NK cells (20, 23), there is no evidence for the effect of hyperthermia on NK cell trafficking. We demonstrated that mEHT facilitated the migration and tumor infiltration of both the primary, *ex vivo* expanded NK cells and the genetically modified, IL-2 producing NK92-MI cells administered subcutaneously one day after loco-regional mEHT treatment of tumor bearing mice. We and others have shown previously that hyperthermia triggered immunogenic cell death accompanied by the release of DAMPs like ATP, HMGB1 and hsp70 which have cytokine-like effects attracting immune cells (2–4, 7, 25, 33). In a similar animal model with C26 colorectal cancer symmetrical allografts in immunocompetent mice Vancsik et al. showed that mEHT triggered a massive infiltration of autologous CD3+ T cells into the tumor (3). In the present study we propose that the mechanism of NK cell recruitment elicited by mEHT consists of multiple cellular events involving DAMP and chemokine secretion as well as extracellular matrix degradation, while NK cell activity is potentiated by increased susceptibility to NK-induced lysis of the target tumor cells. Nagarsekar et al. had proposed that CXC chemokines were a new family of heat-shock proteins. The analysis of the promoters of the CXC genes in both human and mouse showed that they share a common promoter organization with multiple copies of the HSF-1 binding sequence (heat shock response element) demonstrating a strong correlation between activation of HSPs and generation of CXC chemokines (34). In the present study we found that mEHT induced CXCL11 expression in A2058 cells, which has the strongest affinity to its receptor CXCR3, expressed on IL-2 activated primary NK cells and immortalized NK92MI cell line responsible for recruiting effector T cells. Surprisingly, the other two tested chemokines (CXCL9 and -10) were strongly downregulated at this timepoint. In order to gain a more precise overlook on the effect of mEHT on chemokine expression a time course experiment should be performed which then would allow the fine tuning of the therapeutic window of NK infusion depending on the highest peak of chemoattractants elicited by mEHT. Besides HSF1, p53 was also shown to cause tumor cells to secrete various chemokines with the potential to recruit NK cells. Antibody-mediated neutralization of the chemokine (C-C motif) ligand CCL2, but not CCL3, CCL4, or CCL5, prevented NK cell recruitment to the senescent tumors and reduced their elimination (35). It remains to be verified if the activation of p53 by mEHT in the A2058 melanoma contributes or not to NK recruitment *via* the induction of chemoattractant secretion.

Another factor which influences the amount and efficiency of NK cell delivery is the ECM of the tumor. ECM is a dynamic structure, defined by its synthesis by the particular cellular microenvironment and degradation by MMPs which enhance the migration of NK cells into the tumor. The role of hyperthermia in the MMP expression is not well established. It was shown that mild hyperthermia up-regulated matrix metalloproteinases and accelerated basement membrane degradation in experimental stroke (36). In breast cancer the extracellular hsp70 and heat shock protein 90 alpha were identified and shown to activate MMP-2 to facilitate cell migration and invasion (37). In the case of A2058 melanoma MMP-2 and MMP-9 expression was upregulated by various cytokines and inducers (38). We provide evidence that mEHT promotes the expression of MMP-2 and ECM degradation of the A2058 melanoma *in vivo* facilitating the NK cell invasion of the tumor.

Once the NK cells have penetrated the tumor tissue, their cytolytic activity can effectively be manifested only in a permissive tumor microenvironment. Several lines of evidence prove that hyperthermia induces NK cell activity *via* either membrane bound or secreted hsp70 (23). We have previously shown that mEHT upregulated all the three forms (intracellular, secreted and membrane bound) of hsp70 in the B16F10 mouse melanoma (2). In the present study we detected the increase only in the intracellular form using IHC but as it was shown in repeated tumor models, we assume that the membrane bound form is also upregulated, resulting in enhanced cytolytic activity of NK cells. Based on histochemistry and detection of cC-3 expression, we show that the combined therapy results in higher tumor death rate proving the functionality of infiltrated NK cells. We noticed that this effect is more pronounced when mEHT is combined with NK92MI cell line most probably due to its stable expression of the IL-2 transgene rendering it IL-2 independent, while the primary NK cells rely on the host’s IL-2 reservoir.

In summary, based on our results, mEHT might be an effective therapeutic approach in melanoma both *via* its direct inhibition of tumor growth, shown both earlier and here, and through its ability to promote the efficient intratumoral migration and homing of NK-cells. These together may ultimately lead to the complete eradication of melanoma when mEHT is combined with NK-cell therapy. Further preclinical studies using different molecular subclasses of melanomas and NK-cell species are required in combination with mEHT for establishing and efficient clinical translation of our results.

## Data Availability Statement

The raw data supporting the conclusions of this article will be made available by the authors, without undue reservation.

## Ethics Statement

The animal study was reviewed and approved by Hungarian National Scientific Ethical Committee on Animal Experimentation under No. PE/EA/51-2/2019.

## Author Contributions

Experimental model was set up by ABa who also wrote the original draft. The series of experimental tests were performed and analyzed by TV, ABe, AV, IH, DM, and RB. Statistical analysis was done by ABa. The review and editing of the manuscript was done by ZB and TK. All authors contributed to the article and approved the submitted version.

## Funding

This work was supported by the Hungarian National Research, Development and Innovation Office (NVKP_16-1-2016-0042) grant.

## Conflict of Interest

The authors declare that the research was conducted in the absence of any commercial or financial relationships that could be construed as a potential conflict of interest.
